# Problems in Fish-to-Tetrapod Transition: Genetic Expeditions Into Old Specimens

**DOI:** 10.3389/fcell.2018.00070

**Published:** 2018-07-16

**Authors:** Thomas W. P. Wood, Tetsuya Nakamura

**Affiliations:** Department of Genetics, Rutgers, The State University of New Jersey, Piscataway, NJ, United States

**Keywords:** fish, tetrapods, dermal bones, endochondral bones, skull, girdle, fin

## Abstract

The fish-to-tetrapod transition is one of the fundamental problems in evolutionary biology. A significant amount of paleontological data has revealed the morphological trajectories of skeletons, such as those of the skull, vertebrae, and appendages in vertebrate history. Shifts in bone differentiation, from dermal to endochondral bones, are key to explaining skeletal transformations during the transition from water to land. However, the genetic underpinnings underlying the evolution of dermal and endochondral bones are largely missing. Recent genetic approaches utilizing model organisms—zebrafish, frogs, chickens, and mice—reveal the molecular mechanisms underlying vertebrate skeletal development and provide new insights for how the skeletal system has evolved. Currently, our experimental horizons to test evolutionary hypotheses are being expanded to non-model organisms with state-of-the-art techniques in molecular biology and imaging. An integration of functional genomics, developmental genetics, and high-resolution CT scanning into evolutionary inquiries allows us to reevaluate our understanding of old specimens. Here, we summarize the current perspectives in genetic programs underlying the development and evolution of the dermal skull roof, shoulder girdle, and appendages. The ratio shifts of dermal and endochondral bones, and its underlying mechanisms, during the fish-to-tetrapod transition are particularly emphasized. Recent studies have suggested the novel cell origins of dermal bones, and the interchangeability between dermal and endochondral bones, obscuring the ontogenetic distinction of these two types of bones. Assimilation of ontogenetic knowledge of dermal and endochondral bones from different structures demands revisions of the prevalent consensus in the evolutionary mechanisms of vertebrate skeletal shifts.

## Introduction

The fish-to-tetrapod transition during the Devonian is one of the prominent events in vertebrate evolution. The invasion of the land from the water necessitated the evolution of the novel structures in skeletons, musculatures, innervations, visceral organs, and respiratory systems in order to adapt to a terrestrial life (Romer, 1949; Clack, 2012; Dial et al., 2015). Robust appendage skeletons with associated musculatures evolved to support body weight against gravitational force and to aid with movement on land (Coates, 1996; Shubin et al., 2006). To facilitate respiration in air, gill breathing had transformed to lung breathing through morphological innovations (Zheng et al., 2011; Sagai et al., 2017). Sensory systems have been dramatically reconstructed, and the lateral line that sensed physical movement of water was simply lost due to a lack of necessity on land (Piotrowski and Baker, 2014). The function of the kidney shifted from osmoregulation to the excretion of nitrogen in the form of urine or uric acid paste (Mahasen, 2016).

Among the many evolutionary novelties associated with terrestriality, the evolution of the skeleton has attracted much interest from evolutionary biologists. This is mainly because of the ability to trace its evolutionary history through relatively well-preserved structures in the fossil record. Skeletal shifts in the fish-to-tetrapod transition have been understood through the so-called dermal-to-endochondral transition (Westoll et al., 1977; Shubin et al., 2006; Hirasawa and Kuratani, 2015). A fish possesses a significant amount of dermal bones: the dermatocranium, shoulder girdle, jaw, teeth, scales, and fin rays. As lobe-finned fish (sarcopterygians) invaded land, the necessities of these dermal bones for survival fitness largely changed and dermal bones had been reorganized. The dermatocranium of fish skulls, that consists of multiple dermal plates, has experienced major reorganizations in its morphology and composition during the evolution into tetrapods (Coates, 1996; Daeschler et al., 2006). The cranial dermal bones articulate with the shoulder girdle at its posterior end, but, the tetrapod skull has lost several posterior bones, resulting in the dissociation of the skull from the shoulder girdle—the origin of the neck. Dorsal and anal fins are indispensable to stabilize the fish-body for fast swimming in water. However, they are a hindrance on land and have been lost in the tetrapod lineage. Scales that serve as osmotic controls and as protection of the body in an aquatic habitat were also lost during landing. With this concomitant loss of dermal bones, the relative ratio of endochondral bones in vertebrae, girdle, and fins increased in early tetrapods. The endochondral bones in the pectoral girdle enlarged and evolved into the scapula of modern tetrapods (Shubin et al., 2006). In accompaniment with the loss of dermal fin rays in paired fins that propel and balance their bodies, early tetrapods evolved endochondral digits and wrists/ankles (Clack, 2009a; Schneider and Shubin, 2013; Pieretti et al., 2015), helping them to acquire diverse locomotory abilities and extensive maneuverability.

Dermal and endochondral bones are histologically and ontogenetically distinct (Hall, 2005). Dermal bones develop directly from the mesenchyme without a pre-formed cartilaginous model (intramembranous ossification), whereas endochondral bones develop by converting cartilage to bone. The dermoskeleton appears to have originated in the teeth of agnathans such as conodonts and diversified into the dermal skull roof, scales, or fin rays during fish evolution, though evolutionary trajectories of dermal bones are under debate (Donoghue and Sansom, 2002; Sire and Huysseune, 2003; Hirasawa and Kuratani, 2015). Endochondral ossification is hypothesized to have arose much later, in Osteoichthyes (Hirasawa and Kuratani, 2015). Even though a large number of dermal bones have been lost in the transition from fish, tetrapods still possess comparable developmental pathways of dermal bones, which grow calvaria and clavicle bones. The vestiges of the dermal-to-endochondral transition in the developmental programs of vertebrate skeleton cause multiple types of congenital and postnatal skeletal diseases in humans (Gilbert, 2000; Hall, 2005; Wagner and Aspenberg, 2011), such as Progressive Osseous Heteroplasia (POH) or Albright Hereditary Osteodystrophy (AHO) (Regard et al., 2013; Pignolo et al., 2015), which both develop heterotopic dermal bones.

Regardless of the central roles of dermal and endochondral bones in skeletal evolution and clinical cases, scrutiny of the genetic mechanisms that differentiate these distinct types of bones is not enough. With the advent of new technology in molecular biology and paleontology, these problems are now becoming more amenable. The invention of novel sequencing methods promotes comparative genomics with high-throughput output across a diverse range of species (Braasch et al., 2016; Smith et al., 2018). Rapid advancements of genetic manipulation techniques opens a new window to observe functioning of target genes in model and non-model organisms that hold prominent positions in vertebrate evolution (Parker et al., 2014). Furthermore, high-resolution CT scanning is revealing fine details of fossil records and living taxa (Giles et al., 2017; Pardo et al., 2017). These advancements of technology fill in the gaps of understanding evolutionary mechanisms of vertebrates by involving model and non-model organisms into lab-level experiments.

In this article, we summarize the current understanding and problems in the developmental processes and evolutionary shifts of dermal and endochondral bones. First, we review the evolution of cranial dermal bones and the underlying developmental mechanisms. Then, we discuss the skeletal shift from dermal to endochondral bones in the shoulder girdle and current perspectives of the underlying mechanisms. Third, we highlight the approaches that integrate developmental mechanisms into comparative anatomy to answer the fin-to-limb conundrum. Assimilating current knowledge about the molecular mechanisms underlying skeletal shifts of distinct structures would take us one step closer to elucidating the fish-to-tetrapod transition.

## Rearrangements of skull dermal bones

**Figure 1:**
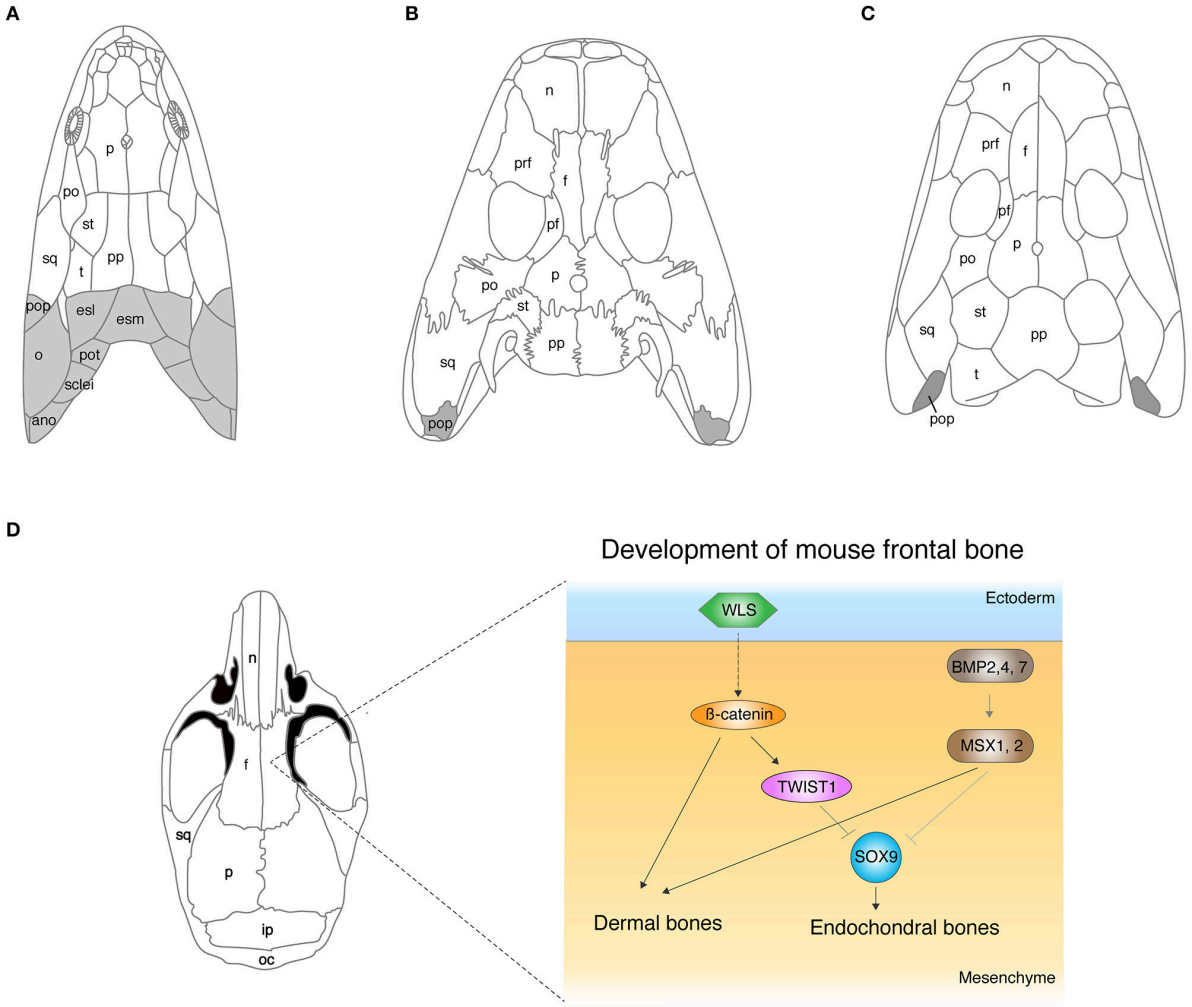
The evolution of cranial dermal bones and the developmental mechanisms. **(A–C)** The skull roof of *Eusthenopteron* **(A)**, *Acanthostega* **(B)**, *Ichthyostega* **(C)**. **(D)** The developmental mechanisms of the frontal bone in mice. The molecules that initiate dermal differentiation from the ectoderm to the mesenchyme have not been identified yet, though the WNT signaling pathway is suggested to be involved (Goodnough et al., 2014). ano, anocleithrum; esl, lateral extrascapular; esm, medial extrascapular; f, frontal; ip, interparietal; n, nasal; o, opercular; oc, occipital; p, parietal; pf, postfrontal; po, postorbital; pop, preopercular; pot, post-temporal; pp, postparietal; prf, prefrontal; st, supratemporal; sq, squamosal; sclei, supracleithrum; t, tabular. Illustrations in **(A–D)** are redrawn with permissions from Clack (2012), Jarvik (1980), and White et al. (2016).

The dermal skull roof is one of the remarkable exemplars for continuous modifications of skeletons during the fish-to-tetrapod transition. The skull roof of sarcopterygians that led to the tetrapod lineage consists of multiple dermal units: such as nasal, parietal (or frontal), temporal, intratemporal, or opercular (Figure 1A). These dermal bones encase endochondral cranial components and protect the primary operative unit of the central nervous system—the brain. The comparative studies of skull morphology between fish and tetrapods highlight apparent distinctions of their broad proportions: the snout is relatively longer, the orbits are located more dorsally, and the skull is flatter in tetrapods. The functional reasons for these skeletal modifications are obscure, but they are likely to be linked with sensory and feeding requirements, and also size of the otic capsules (Clack, 1989). The proportional shifts of skulls from fish to tetrapods are direct consequences of the remodeling of dermal and endochondral bone morphologies, and also simple reductions of dermal bones. *Eusthenopteron* is one of the rhipidistians that lived in shallow freshwater during the late Paleozoic (Andrews and Westoll, 1970). The dermal skull roof of *Eusthenopteron* consists of remarkably large parietals (referred to as “frontals” in actinopterygians) and postparietals (Moy-Thomas and Miles, 1971). Orbits are located at more front of the skull compared with that of tetrapods (Figure 1). *Panderichthys* shares multiple unique features with tetrapods: flat skull, medially located eyes, and no dorsal and anal fins (Schultze and Arsenault, 1985; Boisvert et al., 2008). However, the shape of the four median pairs of cranial dermal bones—nasals, frontals, parietals, and postparietal bones—are rather similar to those of fish. The skull of *Tiktaalik* is also flat with large frontals and postfrontals, which bridges the gap of skull morphology between fish and tetrapods (Daeschler et al., 2006). *Acanthostega*, which has limbs with digits, further fills the gap in our understanding of the difference in the skull roof morphology between fish and tetrapods with its intermediate features—large nasals and frontals, and also the relatively small parietal and postparietal bones (Coates, 1996). In the early tetrapod *Ichtyostega*, nasals and frontals are long, and parietals and postparietals are rather short compared to its early fossil relatives (Jarvik, 1980). Due to these remodeling of dermal bones, the orbits have shifted to a further posterodorsal position—one of the shared features with early tetrapods.

On the lateral side of the skull, gills of osteichthyan fish are covered externally by an opercular series that consists of four dermal bones: the preopercular, opercular, subopercular, and the interopercular. A group of sarcopterygians had adapted to terrestrial life and evolved lungs instead of gills with the gradual loss of opercular series from the skull (Figure 1). Rhipidistians (including *Eusthenopteron*) and *Panderichthys* have operculars and suboperculars on the back of their cheek bones (Figure 1A), whereas *Acanthostega* and *Tiktaalik* barely possess the opercular bone series, but have a rather small preopercular (Coates, 1996; Daeschler et al., 2006; Figure 1B). The skull of *Ichthyostega* also shows a vestige of a preopercular at the back of the squamosals and qudratojugal without any sign of other opercular bone series (Jarvik, 1980; Figure 1C). It remains elusive how much of the function of these preoperculars is for gill breathing or whether they are necessary for other physiological activities in these vertebrates.

Extrascapular series connect the skull to the shoulder bones at the most posterior parts of the skull, and they are another example for the loss of dermal bones in the water-to-land transition (Figure 1). Rhipidistians retain extrascapular series—medial extrascapular and lateral extrascapular—at the posterior of postparietal and tarbular (Andrews and Westoll, 1970). Supracleithrum (girdle bone) articulates to these extrascapular bones via the post-temporal from the posterior side of the skull (Andrews and Westoll, 1970; Figure 1A). However, *Acanthostega* and *Tiktaalik* have lost extrascapular series, resulting in a dissociation of the skull and girdle which led to terrestrial locomotion (Coates, 1996; Daeschler et al., 2006; Figure 1B).

Although completing the details of the skeletal shifts from fish to tetrapods is formidable, the remodeling of the dermal skull roof, and loss of extrascapular and opercular series are general trends with alteration of their habitats across vertebrate phylogeny (Clack, 2012). Of particular note, actinopterygians, including zebrafish which is one of the well-established model organisms, possess comparable opercular and girdle bones to those of the rhipidistians, though genetic programs in teleosts underlying development of these bones might be derived compared with rhipidistians.

## Developmental basis for cranial dermal bones

Along with the extraordinary discoveries of the transitional specimens from water to land, the molecular mechanisms underlying development of cranial dermal bones have been vigorously studied in teeth, operculum, calvaria, mandibule, and other bones (Tucker and Sharpe, 2004; Huycke et al., 2012; Rasch et al., 2016). The dawn of the description of cranial dermal bone development in fish dates back to the early nineteenth century (Pehrson, 1940, 1944, 1958; Aumonier, 1941; Kapoor, 1970; De Beer, 1985; Cubbage and Mabee, 1996). High-resolution histological approaches, including Scanning Electron Microscopy and Transmission Electron Microscopy, have highlighted the developmental process of dermal bones and disparities of structures between dermal and endochondral bones of extinct and living taxa (Verreijdt et al., 2002; Sire and Huysseune, 2003; Witzmann, 2009; Keating and Donoghue, 2016). However, in spite of the continuous efforts (Sire and Huysseune, 2003), the understanding of the development and the evolution of dermal bones still remains poor. Since the changes of cell origins in cranial dermal bone development across species are summarized in previous studies (Piekarski et al., 2014; Hirasawa and Kuratani, 2015; Maddin et al., 2016), we review current perspectives about molecular pathways that are responsible for cranial dermal bone development with the significant emphasis on interchangeability of dermal and endochondral bones.

Dermal bones retain three layers: for example, dentine tubercles are at the outermost surface, middle spongy layer, and basal laminated layer in agnathans (Donoghue and Sansom, 2002). The middle spongy layer is highly vascularized and transports oxygen, proteins, and hormones to support osteogenesis and homeostasis (Percival and Richtsmeier, 2013). Although the details of structures have been modified in different species, the three-layer structure is conserved in vertebrate evolution. The development of dermal bones begins with mesenchymal cell condensations under epithelial layers at their early stages. The aggregated cells directly differentiate into osteoblasts that express osteoblast markers, such as *Runx2* (Abzhanov et al., 2007). In many cases, the epithelial-mesenchyme interaction plays a central role in the initiation of dermal ossification (Sire and Huysseune, 2003). Due to this specific developmental process and the thin structures of dermal bones, surface bones of the fish body consist of dermal bones, not endochondral bones such as scales or spines. Osteoblasts, in turn, induce mineralization around them and differentiate into osteocytes. Distinct from dermal bones, endochondral bones develop from a cartilage template (Long and Ornitz, 2013). The first step of endochondral development is also forming mesenchymal cell condensations, but aggregated mesenchymal cells subsequently differentiate into chondrocytes that express a cartilage marker, *Sox9*. Chondrocytes further differentiate into hypertrophic chondrocytes that induce invasions of blood vessels into developing bones. Chondrocytes are replaced by osteoblasts at the center of long bones, but they do not differentiate into osteoblasts at peripheral regions but rather continue to stimulate cell proliferation and bone growth by Indian Hedgehog (Kronenberg, 2003).

Due to the entrenched idea that dermal bones develop from neural crest cells whereas endochondral bones originate from mesenchymal cells (Smith and Hall, 1990; Sire and Akimenko, 2004), their developmental processes have been hypothesized to be completely distinct. To gain a deeper understanding into the developmental process of dermal bones, Abzhanov and colleagues extensively investigated the gene expression profiles in developing dermal dentary bones of chicken and mouse embryos (Abzhanov et al., 2007). Expanding the previous knowledge of the similarity in some gene expression pattern in dermal and endochondral development including *Runx2*, they discovered that dermal dentary bone expresses some endochondral genes, which had been regarded as specific markers for chondrocytes, *Collagen type 2* and *9*. Moreover, Abzhanov et al. performed fluorescent double *in situ* hybridization that stained different combinations of osteoblast markers and identified four distinct stages in dermal-bone development, including “chondrocyte-like” osteoblasts in the developmental process of dermal dentary bone. These data suggest that the developmental programs of dermal and endochondral bones are in part similar at least in terms of gene expression profiles.

The developmental process of the dermal skull roof (calvaria) has attracted a large amount of attention due to the significance not only in the evolutionary history of vertebrates (Hanken and Hall, 1993; Janvier, 1996), but also in clinical aspects (Tubbs et al., 2012), resulting in rapid advancements of understanding in the developmental mechanisms of cranial dermal bones. Mouse research has considerably contributed to the elucidation of molecular pathways that regulate calvaria development, such as Bone Morphogenetic Protein (BMP), Fibroblast Growth Factor (FGF), Wingless type MMTV integration site family (WNT), Growth and Differentiation Factor (GDF), TWIST, Engrailed 1, Foxc1, and others (Ishii et al., 2015). *Bmp2, 4*, and *7* are expressed in cranial neural crest cells and are one of main factors that develop the calvaria. Conditional knockout of *Bmp2, Bmp4, and Bmp7* in cranial neural crest cells during mouse embryonic development by using the *Wnt-Cre* transgenic mice line resulted in the enlarged frontal fontanelle (Bonilla-Claudio et al., 2012). The conditional double knockout mouse of *Msx1* and *Msx2*, downstream targets of the BMP signal pathway, in cranial neural crest cells, also exhibited severe reduction of frontals, and unexpectedly, newly synthesized cartilage compensated the lack of dermal frontals in the mutant mouse (Roybal et al., 2010). Roybal and colleagues identified the cell source of this ectopic cartilage, which indeed developed from a part of the neural crest cells that do not contribute to dermal bones under normal conditions (Roybal et al., 2010). This data implies a dual role of MSXs in calvaria development—an inducer of dermal bones and suppressor of cartilage bones (Figure 1D). Thus, the level of BMP signaling is likely to be one of main factors that controls the ratio of dermal and endochondral bones through SMAD1/5/8 and MSX which, in turn, induce downstream target genes in cranial bones (Bonilla-Claudio et al., 2012). Abzhanov and colleagues showed that mis-expression of *Bmp4* by virus infection replaces dermal bones by cartilage in chicken frontal bone (Abzhanov et al., 2007), though the result is intuitively opposite to that of the *Msx* conditional knockout mouse. In either scenario, BMP signaling is likely to regulate dermal and endochondral bone differentiation in the concentration-dependent manner. We do not possess any evidence to substantiate the necessities of the exchange program between dermal and endochondral differentiation in mutant mice. The interchangeability between dermal and endochondral development in cranial bones could be the compensatory mechanism to ensure the development of skull bones, yet their contribution to development and evolution is unknown.

The WNT signaling pathway is another major regulator that develops cranial dermal bones and is a balancer for the ratio of dermal and endochondral bones as well (Figure 1D). The conditional knockout mice for β*-catenin*, an intracellular signal transducer of the WNT signal, in cranial neural crest cells and paraxial mesoderm (PAM) by *Engrailed-Cre* transgenic mouse (Goodnough et al., 2012) did not form calvaria. This conditional knockout mouse rather grew cartilage in the original position of calvaria. The phenotype in which dermal bones are replaced by endochondral bones is reminiscent of the conditional double knockout of *Msx1* and *2* and implies that the BMP and WNT pathways crosstalk in calvaria development. Goodnough and colleagues further discovered that β*-catenin* functions to switch differentiation from chondrocytes to osteoblasts *via* TWIST1, which binds to the 3′ untranslated region of *Sox9* mRNA (Goodnough et al., 2012; Figure 1D).

The new study added another key player into the development of cranial bones. Barske et al. identified *Nuclear Receptor 2f* genes (*Nr2fs*) as repressor genes of cartilage development in dermal maxilla in zebrafish (Barske et al., 2018). Expression of *Nr2f* is repressed by *Endothelin-1* in the mandible, resulting in endochondral ossification. Though the function of NRF2 in the limb bud is likely to be different from that of maxilla (Barske et al., 2018), it is fascinating to test NRF2s function in calvaria development. One of the subsequent challenges is elucidating how these distinct signal pathways synergistically or redundantly regulate dermal bone development and how they switch the differentiation between dermal and endochondral bones.

Classical surgical experiments suggested that epithelial-mesenchymal interaction initiates dermal bone development in the mesenchyme, yet the molecular mechanisms had remained elusive for a quarter of a century (Hall, 2005). Goodnough and colleagues deployed a conditional knockout system to answer how the epithelial layer promotes development of dermal bones in the cranial mesenchyme (Goodnough et al., 2014); the conditional deletion of *Wls*, the trafficking regulator of the WNT ligand, by *Crect* (ectodermal Cre) mice showed the entire loss of the calvaria bones. However, the conditional knockout of *Wls* in the underlying mesenchyme by *Dermo1Cre* (*Cre* expression in cranial mesenchyme and meningeal progenitor cells) did not show a comparable dermal phenotype (Goodnough et al., 2014). These data suggest that the WNT signal from the ectoderm, presumably through diffusion of proteins, initiates cranial dermal bone development in the underlying mesenchyme. Because multiple WNT ligands are expressed in the epithelial layer and cranial mesenchyme, it is time-intensive to identify the major WNT ligand that transmits signal from the ectoderm to the mesenchyme (Goodnough et al., 2014). While many genes have been discovered to be responsible for cranial dermal bone development, understanding the initial mechanisms that initiate dermal differentiation—the upstream of the WNT signal in the ectoderm—would be one of next challenges. Further experiments are expected to identify molecules or any other cues that trigger the differentiation of cranial dermal bones at the upstream of the WNT signal pathway.

Along with the discoveries of many genes that develop cranial dermal bones, actual genetic loci that have contributed to vertebrate cranial evolution is still obscure. The BMP and WNT signals, which are necessary for the development of the mouse calvaria, are also involved in developing and diversifying skull shapes in fish (Albertson et al., 2005). The inhibition of WNT signaling by a chemical agonist or antagonist in cichlids created the diversity of preorbital morphologies, which phenocopy cichlid facial diversity (Parsons et al., 2014). The detection of single nucleotide polymorphism (SNP) in β*-catenin* of cichlids also supports the involvement of the WNT signal in facial diversity (Loh et al., 2008). Further assessments to test the functional roles of the BMP and WNT signal pathways in development of the fish skull would enrich developmental and evolutionary basis of fish skull diversity. During the fish-to-tetrapod transition, many cranial dermal bones are lost or reorganized (Clack, 1989; Daeschler et al., 2006). It is likely that different genetic loci contributed to the modifications of different bones, and/or multiple genetic loci contributed to a rearrangement of a single bone, as we have observed in complex evolutionary traits of other vertebrates (Linnen et al., 2013). To gain complete pictures of the molecular mechanisms underlying the evolution of cranial dermal bones, strategies that comprehensively identify genetic loci which contribute to specific phenotypes are ongoing and are successfully capturing responsible regions (Jones et al., 2012; Parsons and Albertson, 2013; Miller et al., 2014). The contribution of genetic loci, which are identified by these QTL studies in closely related species, to a more long-time scale evolution of vertebrates (i.e., the fish-to-tetrapod transition) remains elusive.

## Disconnection of the shoulder girdle from the skull

**Figure 2:**
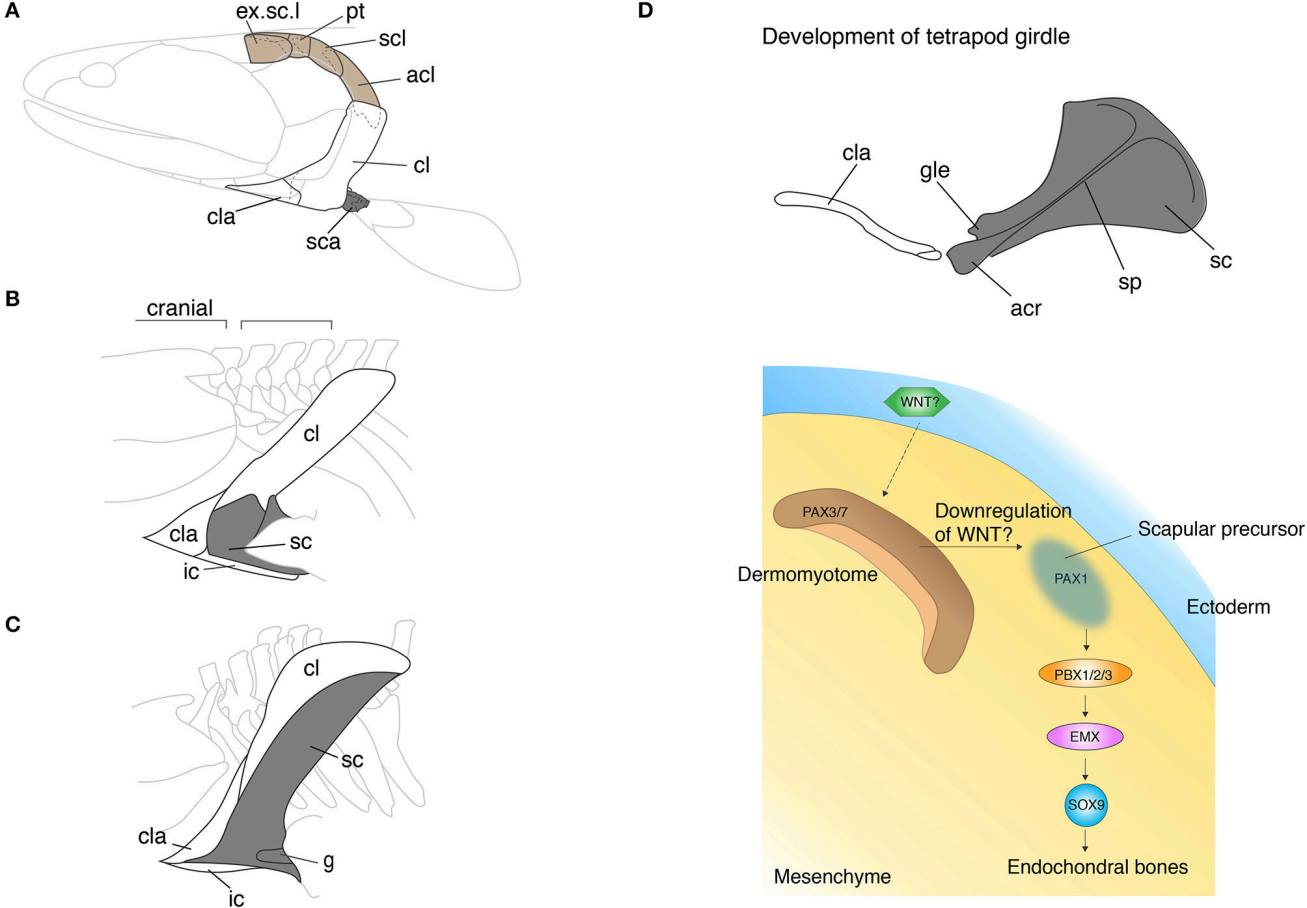
The skeletal shift from dermal to endochondral bones in the pectoral shoulder girdles. **(A–C)** The pectoral girdles of *Eusthenopteron* **(A)**, *Ichthyostega* **(B)**, *Eryops* **(C)**. Brown shaded bones (extrascapular, post-temporal, supracleithrum, and anocleithrum) in **(A)** have been lost during the fish-to-tetrapod transition. Shaded bones depict endochondral bones. Note that the endochondral bones have enlarged with the concomitant decrease of the dermal bones. In **(B,C)**, the spaces between the skull and pectoral girdle demonstrate the origin of the neck. **(D)** The developmental mechanisms of the scapula in mouse and chicken embryos. The epithelial-mesenchymal transition produces prospective scapular cells from the dermomyotome. The understanding of developmental programs underlying the dermal bone development is still poor (see text). acl, anocleithrum; acr, acromion; cl, cleithrum; cla, clavicle; g, glenoid fossa; ic, interclavicle; sc, scapula; sca, scapulocoracoid; scl, supracleithrum; sp, spine; Illustrations in **(A–C)** are redrawn with permissions form Andrews and Westoll (1970), Jarvik (1980), and Gregory (1951). The illustration of the mouse scapula is adapted with permission from Kuijper et al. (2005).

The pectoral girdle morphologies of fish and tetrapods are spectacularly diverse. Since the girdle links appendicular bones to the body trunk and serves as the base of attachment for muscles of the neck and pectoral appendages, the girdle holds a critical position in the evolution of vertebrate locomotion. Comparison of pectoral girdle morphology across vertebrates highlights the general trend in girdle evolution—the reduction of dermal bones and enlargement of endochondral bones. In fish, the series of pectoral girdle bones mainly consists of dermal bony plates: the supracleithrum, postcleithrum, cleithrum, and the clavicle along the dorsoventral axis (Andrews and Westoll, 1970; Figure 2A). The supracleithrum articulates the pectoral girdle series to the post-temporal (Figure 1A) and orchestrates movements of the head and paired pectoral appendages. Also, the fish pectoral girdle has the scaupulocoracoid, a relatively small endochondral bone that connects the humerus to the pectoral girdle via the glenoid fossa. To invade land, early vertebrates required a robust skeleton to support their body weight without buoyancy. During the fish-to-tetrapod transition, dermal bones had become reduced and simultaneously the endochondral scapulocoracoid had enlarged (Figures 2A–C). The scapulocoracoid had split into the scapula and procoracoid bones, which both develop from different developmental centers. In amniotes, the shoulder girdle has been further modified and it has become three bones: the scapula, the procoracoid, and the coracoid.

The developmental processes of girdle bones (Grandel and Schulte-Merker, 1998; Davis et al., 2004; Sears, 2004; Pomikal and Streicher, 2009; Boisvert et al., 2013; Sears et al., 2013; Dillman and Hilton, 2015; Warth et al., 2017) and the associated muscles (Ericsson et al., 2013; Diogo et al., 2014; Masyuk et al., 2014; Pu et al., 2016) have been described in many taxa, yet the problematic evolutionary history of girdle bones has hampered us from understanding the cellular origins of girdle bones. Kague and colleagues tested whether neural crest cells contribute to girdle bones in zebrafish by using *Wnt1-Cre* transgenic fish and confirmed that neural crest cells do not migrate into dermal girdle bones including the supracleithrum, postcleithrum, cleithrum, and the endochondral scapulocoracoid. This finding raises a possibility that the zebrafish girdle bones originate from the lateral plate mesoderm (LPM) or the PAM, yet these hypotheses have not been tested. The cell origins of girdle bones, particularly the scapula, have been more intensively investigated in tetrapods: salamander, turtle, chicken, and mouse embryos (Burke, 1991; Huang et al., 2000; Malashichev et al., 2008; Valasek et al., 2010; Kague et al., 2012; Nagashima et al., 2016). All of these studies support the ontogenetically conserved pattern across tetrapods. The tetrapod scapula has, at the very least, a dual origin—the LPM and somites. Piekarski and Olsson transplanted GFP-labeled somites to wild-type axolotl and demonstrated the somitic contribution to the suprascapular (Piekarski and Olsson, 2011). Burke performed the removal of somites adjacent to the fore limb region of turtle embryos, which resulted in scapular defects (Burke, 1991). Huang and colleagues performed chick-quail chimeric analyses and revealed that the head and neck of the scapula originates from the LPM, but the scapula blade develops from the dermomyotome of somite 17–24 (Huang et al., 2000). Intriguingly, the cells maintain their topology of original somites in the developing scapular such that muscles attach to a specific position of the scapula blade that originate from same somites. The developmental logic underlying the dual origin of scapular cells is explained by Piekarski's non-canonical “position-dependent” hypothesis that proposes that the scapula develops from its most adjacent tissue, either somite or LPM (Piekarski and Olsson, 2011). Mouse studies also showed that the mouse scapula originates from somites by using *Pax3-Cre* transgenic mice (Valasek et al., 2010). In addition, Matsuoka et al. showed that neural crest cells contribute to the scapular spine, coracoid, and acromion by using *Wnt1-Cre* lineage trace system in mice (Matsuoka et al., 2005). Further research could test the contribution of neural crest cells into the girdle bones of primitive actinopterygian or elasmobranchs; whether non-neural crest origin of zebrafish girdle is an evolutionary conserved pattern in fish lineage.

Knowledge about the developmental programs of fish girdle bones is still fragmented. *Syu* homozygous zebrafish that have a mutation in the *Sonic hedgehog a* coding sequence showed a severe defect in cleithrum and pectoral fin development (Neumann et al., 1999). The morpholino knockout of T-box gene, *Tbx5*, also affected the pectoral fin module; with the loss of pectoral fins, the scapulocoracoid, postcoracoid process, and the cleithrum were also severely affected (Ahn et al., 2002). Further, *Dlx5a* and *Dlx6a* morpholino-mediated knockdown in zebrafish disrupted not only the pectoral fin, but also girdle bone development (Heude et al., 2014). These findings indicated that the development of fins and girdles, either dermal or endochondral, are interlinked by the same genes and cannot be simply separated because of their close topology in the developing body.

In tetrapods, especially in chickens and mice, more genes have been uncovered in scapula and clavicle development (Huang et al., 2006; Figure 2D). The cranial part of the pectoral girdle, including the acromion, coracoid process, and the glenoid fossa, develops from the LPM. The developmental programs of the anterior scapular bone shares the same set of genes with the limb bud as we have seen in the development of the zebrafish pectoral girdle; *Dlx* (Heude et al., 2014), *Islet1* (Itou et al., 2012), *Tbx5* (Valasek et al., 2011), and *Twist1* (Krawchuk et al., 2010; Loebel et al., 2012) affect both girdle and limb development. Contrary to the canonical hypothesis, the scapular blade develops from dermomyotome, not sclerotome, which goes through the epithelial-mesenchymal transition (EMT) (Figure 2D). Several transplantation studies in chicken embryos showed that signals from the ectoderm to dermomyotome are necessary for this EMT (Malashichev et al., 2005). The molecules that induce EMT in the dermomyotome from the ectoderm have not been identified, yet the attenuation of the WNT signal is likely to be involved. Moeller and colleagues ectopically expressed Carboxypeptidase Z (CPZ), which possesses a WNT binding domain, in somites of chicken embryos and discovered that the WNT signal downregulates *Pax1* expression that is necessary for scapular development and promotes *Pax3/7* expression that is necessary for limb muscle development (Moeller et al., 2003). WNT6 in the ectoderm was suggested as a primary diffusible ligand to maintain *Pax3/7* expression, nevertheless further experiments to verify its function in the EMT are imperative (Schmidt et al., 2004). Once the EMT produces prospective scapular cells with *Pax1* expression, *Pbx* family genes become key players to regulate and pattern scapular development (Figure 2D). PBX1/2/3 are expressed in the proximal limb bud and promote cartilaginous condensation through binding with EMX2 (Capellini et al., 2010, 2011). In parallel with developing cell condensation, PBX and BMP (Hofmann et al., 1998; Capdevila et al., 1999) regulate *Hoxa5* and *Pax1* (Timmons et al., 1994; Hofmann et al., 1998; Aubin et al., 2002) to pattern the acromion and the scapular head. The patterning of posterior scapula is established by *Alx1* (Capellini et al., 2010), *Tbx15*, and *Gli3* (Kuijper et al., 2005) that are also downstream of PBX1.

Despite of the discovery of a number of genes for scapular development, little is known about the molecular mechanisms underlying the development of the dermal clavicle in the shoulder girdle. Kuijper and colleagues investigated the girdle phenotype of triple knockout mice of *Alx4, Cart1*, and *Tbx15* and discovered that the clavicle showed a severe phenotype while the scapula showed a minorly affected morphology in these mutant mice (Kuijper et al., 2005). These data suggest that these genes may regulate more or less specifically dermal bone development in the shoulder girdle, but the precise mechanisms are unknown.

Fish girdle bones are almost all dermal bones, which may utilize epithelial-mesenchymal interaction or ossify by themselves without any input from epithelial tissue. The ectodermal signal in the scapular development of tetrapods is most likely important to differentiate the competent cell population for the prospective shoulder girdle, not to trigger the bone developmental program itself. As reviewed above, in the shoulder girdle, the developmental programs of dermal and endochondral bones are presumably intermingled; the genes affecting endochondral bone development also affect dermal bone development in most cases. This data implies that the cell sources and developmental programs for these two types of bones in the shoulder girdle are not obviously separated due to their complicated evolutionary history—between the skull and body trunk. It is compelling to test how the epithelial-dermomyotome interaction is conserved in the development of the scapulocoracoid, and dermal bones of fish.

## Fins into limbs

**Figure 3:**
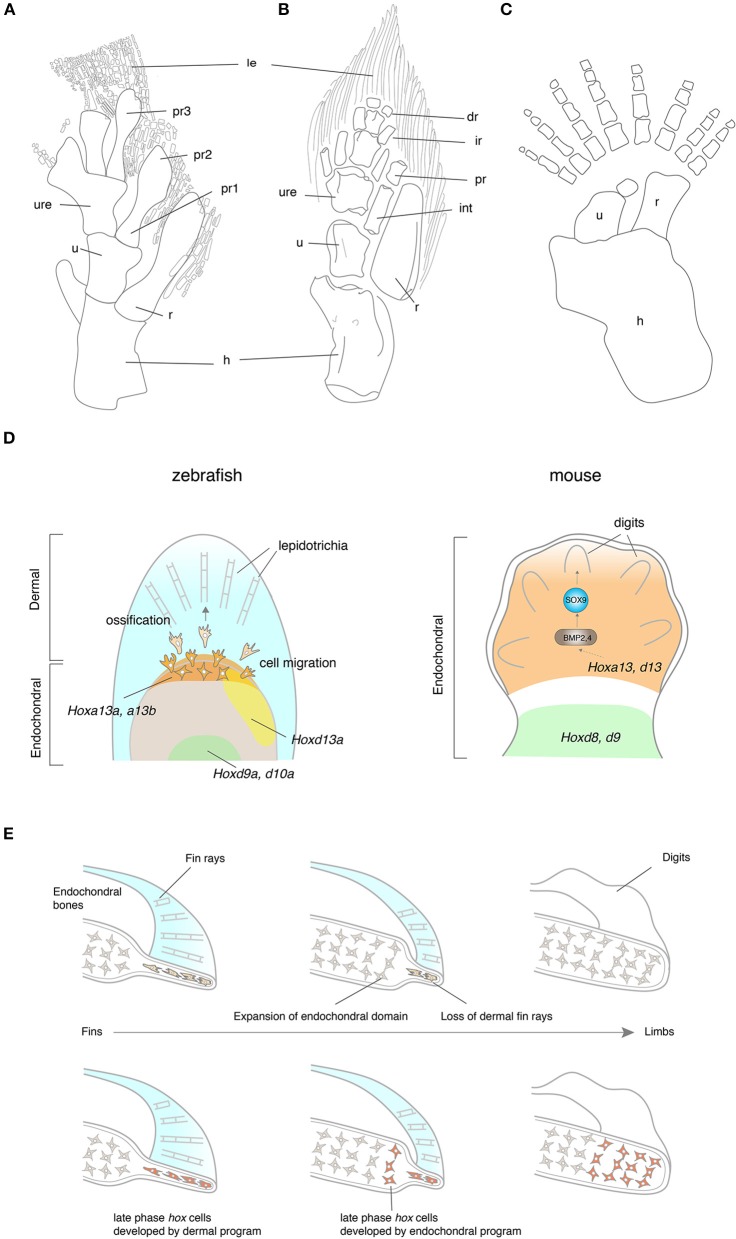
The fin-to-limb transition and their developmental basis. **(A–C)** The pectoral fins and limbs of *Eusthenopteron* **(A)**, *Tiktaalik* **(B)**, and *Acanthostega* **(C)**. Note that *Eusthenopteron* and *Tiktaalik* possess distal fin rays with the endochondral skeletons. **(D)** The developmental mechanisms of fin rays and digits. In zebrafish, *hox13* genes are expressed in a distal domain of the endochondral disk. The cells that experienced the late phase *hox* activity migrate out from the distal endochondral disk into the fin fold, that differentiate into the lepidotrichia. In mice, *Hox13* genes are expressed in the autopod, which develop digits at a later stage. HOXA13 is suggested to bind the regulatory region of *Bmp2* and *4* by ChIP experiments (Knosp et al., 2004). While many of the downstream genes of HOX13 have been explored in mice, genes regulated by HOX13 in fish have not been identified. Note that the ossification of lepidotrichia and digits takes place at a later stage than the expression of *Hox13* genes. **(E)** Hypotheses for the fin-to-limb transition. The fin rays (cells shaded by light orange) have degenerated and the endochondral domain (cells shaded by light gray) expanded during the appendage evolution (top). This hypothesis supports that digits and wrists are novel domains that have been acquired as fish have evolved to tetrapods. Another hypothesis claims that the fish fin has an antecedent of digits and wrists (bottom). The cell histories between fish fin rays and tetrapod digits are comparable in terms of their *hox* expression pattern during the embryonic development (cells shaded by orange). In this hypothesis, the cell differentiation program of fin rays might have changed to become endochondral bones, resulting in the acquisition of digits and the wrist. dr, distal radials; hu, humerus; int, intermedium; ir, intermediate radials, le, lepidotrichia; po, postaxial process, pr, preaxial radials; pro, proximal radials; ra, radius; ul,ulna, uln, ulnae; Illustrations of **(A–C)** are redrawn with permissions from Andrews and Westoll (1970), Shubin et al. (2006), and Coates (1996).

The evolution of tetrapod limbs from fish fins is one of the most remarkable transitions in vertebrate history (Clack, 2009a,b). Whereas fish fins consist of endochondral bones in a proximal domain and dermal fin rays in a distal domain, tetrapod limbs are exclusively composed of endochondral bones. In *Eusthenopteron*, the scapulocoracoid articulates with the humerus that further connects to the ulna and the radius (Andrews and Westoll, 1970; Figure 3A). The distal ends of the ulna and the radius attach to the preaxial radials, which are followed by the lepidotrichia. The pectoral fin of *Tiktaalik* presents an intermediate structure between fish and tetrapods with regards to its morphology and function. The *Tiktaalik* fin possesses elaborated distal endochondral bones; their morphology and mobility is reminiscent of distal appendages of tetrapods (Shubin et al., 2006; Figure 3B). In contrast to the extension of endochondral domain toward the distal direction, the dermal fin rays of *Tiktaalik* are much reduced compared with that of fish. A further transition from fins into limbs is observed in *Acanthostega*. The paired appendages of *Acanthostega* retain comparable digits to those of tetrapods with a stout humerus, ulna, and radius, but without any evidence of fin rays (Coates, 1996; Figure 3C). The number of digits is eight in the fore limb of *Acanthostega*, but *Ichthyostega* shows a reduction of digit number toward five, which is the shared feature with later tetrapods—pentadactylism (Jarvik, 1980). The reduction of dermal bones and the increase of endochondral bones in the evolution of paired appendages is a similar trend such as when shoulder girdle bones evolved and this trend is significantly associated with the functional importance of the endochondral appendage in terrestrial life. However, it is not understood whether underlying molecular mechanisms of shoulder and limb evolution are common, or if they employ distinct mechanisms.

Since fish lepidotrichia, one of the major components of fin rays, directly ossifies without a cartilage model, the cell origin of fin rays had been assumed to be neural crest cells like other dermal bones, such as scales. However, the recent genetic labeling of the specific cell population in zebrafish revealed the other cell origins of fin rays. Lee and colleagues used *Tbx6 promoter-Cre* transgenic fish to trace the derivatives of PAM and discovered that the PAM solely contributes to the development of fin rays in the caudal fin (Lee et al., 2013). Another line of evidence from medaka also supported this conclusion (Shimada et al., 2013). Shimada and colleagues transplanted somite cells of transgenic fish that ubiquitously expresses DsRed into that of wild-type fish and confirmed that these red fluorescent cells contribute to median fin rays. In parallel with this transplantation experiment, they proved the contribution of the PAM to median fin rays by “IR-LEGO” in which *Cre-loxP* system irreversibly marks target cells with heat shock stimulus. Deploying the same labeling system, they discovered that the LPM, not neural crest cells, contributes to lepidotrichia formation in the pectoral fin (Yano et al., 2012; Shimada et al., 2013). Therefore, model organisms, zebrafish and medaka, provide us the ability to perform genetic experiments to test our hypotheses, yet, another compelling experiment is to test the evolutionary synapomorphy with primitive actinopterygians. Tulenko and colleagues injected DiI into the LPM shortly after gastrulation and confirmed that DiI-labeled LPM cells contribute to pectoral fin fold (Tulenko et al., 2017). The distinct cell origins of lepidotrichia (PAM or LPM) in unpaired and paired fins refuels the discussion of the origin of paired fins, which share same *Shh* cis module with unpaired fins (Freitas et al., 2006; Letelier et al., 2018). Mice and chicken studies have provided us with comparable knowledge of the cellular origins of the limb bud to that of fish. Gros and Tabin demonstrated that the epithelial cells of the somatopleural LPM contribute to the limb bud mesenchyme through EMT (Gros and Tabin, 2014). Further, the genetically labeled mesenchymal cells which expressed *HoxA13* exclusively contribute to digits and wrist bones (Scotti et al., 2015). This evidence collectively suggests that LPM-derived cells express *HoxA13* and contribute to endochondral bones, yet no scrutiny of cell origins of *HoxA13* cells have been conducted to date.

The genetic underpinnings of the development of paired appendages have been profoundly investigated in the embryonic limbs of chickens and mice (Zeller et al., 2009). *Hox* genes—Homeodomain-containing transcription factors—play pivotal roles in body patterning during embryonic development and are expressed in the limb bud (Zakany and Duboule, 2007). In early development of the limb bud, the genes in *HoxA* and *HoxD* clusters are expressed in a nested manner from the posterior to anterior limb bud; expressions from “anterior” genes (3′ side genes of *Hox* clusters. e.g., *Hoxa1* or *Hoxa2*) of *HoxA* and *HoxD* clusters are relatively broader than that of “posterior” genes (5′ side genes of *Hox* clusters. e.g., *Hoxa13* or *Hoxd12*) to the anterior domains of the limb bud. According to this nested expression pattern, *Hox* genes produce positional information along the anteroposterior axis inside the limb bud. *Sonic Hedgehog* is expressed at the Zone of Polarizing Activity (ZPA) and constitutes a positive feedback loop with *Hox* genes, resulting in cell proliferation and also providing positional information with *Hox* genes (Zeller et al., 2009) in the mesenchyme. At later developmental stages, anterior *HoxA* and *D* expression is limited in a proximal domain of the limb bud, while posterior *HoxA* and *D* genes gain a new expression domain in the autopod (Figure 3D). The functional roles of these site-specific *Hox* expressions were tested in knockout mice. The *Hoxa13* and *d13* combinatorial knockout mice lost the entire autopod (Fromental-Ramain et al., 1996b), while the *Hoxa9* and *d9* knockout mice eliminated the humerus without affecting autopod development (Fromental-Ramain et al., 1996a). Recent studies revealed genomic underpinnings of the two-phase *Hox* expression; the long-range contacts of the regulatory regions to *HoxA* and *D* clusters from centromeric side or telomeric side establish 3D chromatin loop structures (Topologically Associating Domain, TAD) (Montavon et al., 2011; Andrey et al., 2013) and assure physical proximity of the enhancers to *Hox* genes. These large chromatin structures regulate a group of target gene expression in a time- and site- specific manner and develop two distinct domains in the limb bud—the proximal limb (the humerus, ulna and the radius) and the distal limb (digits).

*Hox* genes are expressed in a nested manner in the endochondral disk of pectoral fins of fish as well (Ahn and Ho, 2008). At early stages, expression of “posterior” *hoxa* and *d* genes are more restricted to a posterior domain of the fin bud compared with that of “anterior” genes. At later stages, expression from posterior *hoxa11-13* and *d11-13* is restricted to a distal domain of the fin bud as “posterior genes” of mice in the limb bud (Figure 3D). Woltering et al. and Gehrke et al. revealed that the chromosomal topologies underlying these two-phase *hox* expression patterns are the shared feature with that of mouse by 4C-sequencing, suggesting that fish fins already retain a dual TAD system in paired fins before evolving digit and wrist (Woltering et al., 2014; Gehrke et al., 2015). Other major genetic networks for the development of paired appendages are also peculiarly conserved between the endochondral disk of fish and limb bud of tetrapods: *Tbx5* (Ahn et al., 2002; Adachi et al., 2016), *Shh* (Neumann et al., 1999), *Fgf8* (Jovelin et al., 2007), *Bmp2* (Laforest et al., 1998), and others. During the fin fold development, the LPM migrates from the proximal fin bud into the distal fin fold, and the cell configuration becomes flat and thin in the distal domain (Thorogood, 1991; Yano et al., 2012). Concomitant with physical cell migration, *hoxa13a* expression also migrates out from the endochondral disk to the proximal fin fold (Ahn and Ho, 2008; Nakamura et al., 2016; Tulenko et al., 2016). Surprisingly, during the late development of fin fold, the gene expression profile of the fin fold in paddlefish and of the autopod in mice is rather similar despite their distinct histological structures (Tulenko et al., 2017). *Shh* is expressed at the posterior edge of the pectoral fin fold, and *Fgf10* and *hoxa13* is in the broad domain of the fin fold, all of which are reminiscent of gene expression patterns of the mesenchyme in the mouse limb bud (Tulenko et al., 2017).

The shared developmental programs between the fin fold and the autopod inevitably drive further questions. For example, how do these two appendage primordia develop into distinct dermal or endochondral bones from the conserved gene expression patterns? The signaling pathways underlying the development of dermal fin rays has been vigorously studied in normal development and also in the regeneration process after amputations of fins (Wehner and Weidinger, 2015). As the fin fold develops, actinotrichia form at a distal tip and leads lepidotrichia development at its proximal part (Wood and Thorogood, 1984; Durán et al., 2011). *Actinodin* (*And*) *1* and *2* serve as non-collagenous components of actinotrichia in fin development, but they had been lost from the genome of the tetrapod lineage (Zhang et al., 2010; Figure 3D). Zhang and colleagues tested their function in zebrafish by morpholino-mediated knockdown, resulting in the loss of fin rays from the pectoral fin. Given that these genes were lost from the tetrapod genome, they posited that the loss of *And* genes from the fish genome is likely to have promoted the fin-to-limb transition through the loss of lepidotrichia (Zhang et al., 2010; Lalonde et al., 2016). Currently, the analysis of regulatory mechanisms of *And1* expression is ongoing (Lalonde et al., 2016). With the developmental pathways of fin rays, the mechanisms that initiate dermal fin ray differentiation in the fin fold remain enigmatic. The WNT and SHH signal from the epithelial cells stimulates cell differentiation during the regeneration of fin rays, but the genetic mechanisms that induce the differentiation of dermal bones in normal fin rays remain unknown (Quint et al., 2002; Wehner and Weidinger, 2015). Harris et al. screened *eda* (ectodysplasin) mutant fish, which show the drastic loss of dermal fin rays from paired and unpaired fins as well as the phenotypes of other dermal bones such as the loss of scales (Harris et al., 2008). Since *eda* is expressed in the epidermal placode and *edar* (ectodysplasin receptor) is in the basal cells of the forming placode in scale development, the EDA signaling pathway is likely to also be involved in fin ray development through epithelial- mesenchymal interaction. Though it is a widely shared consensus that the apical fold [AF-the epithelial structure that forms after Apical Ectodermal Ridge (AER)] plays a critical role in fin fold development (Yano et al., 2012), little is known about how the AF interacts with the underlying mesenchyme and induces dermal ossification.

Due to the apparent loss of the autopod domain in *Hoxa13* and *Hoxd13* double knockout mice (Fromental-Ramain et al., 1996b), the function of HOX13 has been one of main foci in understanding the endochondral development of the limb bud (Figure 3D). The overexpression of HOXA13 in the limb bud alternated cell-cell adhesion and affected the size of cartilage condensation (Yokouchi et al., 1995). Knosp et al. identified *Bmp2* and *Bmp7* as direct targets of HOXA13 by using a ChIP assay (Knosp et al., 2004; Figure 3D). BMP signaling is well known to play key roles in cartilage and bone development, identities of digits, and interdigital apoptosis during limb development (Suzuki, 2013). Thus, they are likely to be directly involved in the differentiation process of endochondral bones at the downstream of HOXA13. HOXD13 is also suggested to bind the regulatory regions of *Sfrp1, Barx1*, and *Fbn1*, all of which are indispensable for normal skeletogenesis (Salsi et al., 2008). Recent advancements of technology in genomics have further accelerated extensive identification of the downstream genes of HOX13 groups. Whole genome tiling arrays and RNA-sequencing explored whole gene expression profile including non-coding RNA in wild-type and *Hox*9-11 mutant mice (Gyurján et al., 2011; Raines et al., 2015). These experiments successfully identified a number of *Hox* downstream genes, including bone developmental factors such as *Runx*, in the limb development. ChIP-sequencing of HOXA13 and HOXD13 in cultured cells of chicken and mouse limb buds suggested that HOX13 and CTCF co-bind to a number of genomic loci (Beccari et al., 2016; Jerković et al., 2017). Given that the CTCF transcription factor regulates 3D chromatin structures, this finding implies that HOX13 transcription factors not only bind directly to their target sites, but rather regulate chromatin structures broadly with CTCF, which consequently shifts the broad gene expression profile.

In parallel with the elucidation of the developmental programs of dermal fin rays and endochondral bones in fish, the evolutionary mechanisms from fins into limbs have been closely investigated (Sordino et al., 1995; Woltering and Duboule, 2010; Schneider and Shubin, 2013; Yano and Tamura, 2013; Woltering et al., 2014; Onimaru et al., 2015; Tanaka, 2018). Sordino et al. suggested that digits and wrists are novel domains of tetrapods due to a striking difference in the expression pattern of *Hoxa-11* between fish and tetrapods (Sordino et al., 1995). Woltering et al. injected a *tetraodon* BAC vector containing *hoxa13b* region into mice and observed the expression pattern of *hoxa13b*, which is regulated by fish regulatory domains in mouse limbs. They observed that the expression domain of the fish *hoxa13b* is confined to a proximal domain of the limb bud of mice, not in a distal domain like mammal *Hoxa13* (Woltering et al., 2014). Woltering et al. also state that while fish have most of the necessary genes and regulatory architecture indispensable to form digits, 5′ regulatory landscapes in fish cannot specify a distinct digit territory (Woltering et al., 2014). This suggests that distal pectoral fins and distal limb buds are not comparable in a classical sense of homology as there is a lack of a common ancestral structure. Freitas et al. overexpressed *hoxd13a* in order to investigate the function of 5′ *hoxd* expression in fin development and discovered that the overexpression of *hoxd13a* results in the proliferative expansion of chondrogenic tissue distally that is akin to autopod development (Freitas et al., 2012). Leite-Castro et al. also propose possible mechanisms of the fin-to-limb transition, a consequence of various modifications in *HoxA* genes, such as: expansion of polyalanine repeats within the HOXA11 and HOXA13 proteins, an acquisition of novel ncRNA with an inhibitory function of HOXA11 or *cis*-regulatory evolution of *hoxa13* (Leite-Castro et al., 2016).

Contrary to the entrenched idea that dermal bones and endochondral bones are ontogenetically and histologically distinct, recently new evidence has implied a possible ontogenetic interchange between these two types of bones in appendage evolution. First, the development of dermal fin rays is unique; the gene expression profile during their development is at an intermediate state between endochondral and dermal bones in appendage evolution. *Col2a1* and *Col10a1*, which are regarded as specific markers of endochondral bones, are expressed despite the absence of cells stained by cartilage staining in fin rays (Smith et al., 2006). Second, functional analyses of *hox13* genes provide a new insight for development of the fin fold. Lalonde and Akimenko deleted *hoxa13a/hoxd13a* expressing cells and observed the defects in the formation of fin rays (Lalonde and Akimenko, 2018). Double knockout zebrafish of *hoxa13a* and *a13b*, and triple knockout fish of *hoxa13a, a13b*, and *d13a* lost dermal fin rays (Nakamura et al., 2016). These data demonstrated that *hox13* genes are indispensable for fin ray development, whereas *hoxa13* and *d13* are necessary to develop endochondral digits in mice. Third, double knockout zebrafish of *hoxa13a* and *a13b*, and triple knockout fish of *hoxa13a, a13b*, and *d13a* increased the number of distal endochondral bones along the proximodistal axis with the loss of dermal fin rays (Nakamura et al., 2016). Also, the repetitive excisions of the AF, which is critical to develop fin rays, from the developing pectoral fin, extended the size of the endochondral disk distally (Yano et al., 2012). Summarizing all data leads us to a novel hypothesis—the developmental program between dermal and endochondral programs are interchangeable and the dermal genetic network has been replaced by the endochondral network in appendage evolution (Nakamura et al., 2016; Tulenko et al., 2016, 2017; Paço and Freitas, 2018; Figure 3E). Given that LPM cells contribute to lepidotrichia, LPM cells that express *hoxa13* genes are most likely to differentiate into dermal fin rays in fish, whereas LPM cells that experience *hoxa13* and *d13* develop endochondral digits in tetrapods. Further dissection of the cell origins and fate mapping of *Hoxa13* cells in the fish fins and tetrapod limbs would provide us with more insights for the mechanisms of the fin-to-limb transition.

Though the genetic mechanisms underlying for interchanges between dermal and endochondral bones remain elusive, gradual losses of gene expression that are indispensable for the development of fin rays such as *And1*, are likely to play roles (Zhang et al., 2010). Masselink and colleagues' research would illuminate a path to approach the underlying mechanisms. They discovered that whereas ectodermal cells develop an AER which promotes tissue growth via *Fgf8* and *Wnt3* in tetrapods (Lewandoski et al., 2000; Barrow, 2003; Boulet et al., 2004), somitic cells contribute to the AF development (Masselink et al., 2016). They removed somite-derived cells by a genetically targeted cell ablation system before the AF induction, resulting in the severe disruption of AF development, as well as a lack of actinotrichia deposition. Furthermore, they marked somite-derived cells via the introduction of the photoactive protein Kaede, and induced apoptosis by laser illumination. The removal of the somite-derived cells from the AF significantly decreased the size of the actinotrichia as well as a reduction by 30% in the length of the fin fold. They concluded that the swap of the cell contributions from somitic cells to ectodermal cells in the AF was likely to drive the evolutionary shift from the AF to the AER and, consequently, lead to the fin-to-limb transition. Extensive investigations and comparative studies of the cell lineages that contribute to the AF, AER, fin fold, and the endochondral disk would shed light on the evolutionary trajectories of appendages.

## Genetics and genomics into old questions

Endless discoveries in paleontology have led to continuing scientific questions in vertebrate evolution. The skeletal shifts between dermal and endochondral bones are examples of major vertebrate transitions from water to land, yet revealing the genetic mechanisms underlying their evolution is a long endeavor in evolutionary biology. Integration of novel techniques in both molecular biology and paleontology would accelerate the understanding in developmental and evolutionary mechanisms of cranial dermal bones, shoulder girdles and appendages during the fish-to-tetrapod transition.

In contrast to the canonical hypotheses, new studies demonstrated that the cell origins of some dermal bones are obviously from the LPM and the PAM, not neural crest cells. Furthermore, developmental programs for dermal bones and endochondral bones are ontogenetically interchangeable in skull development by deploying distinct cell origins. They may be interchangeable even in a single cell population of appendage development, though further assessment is necessary. These new discoveries make our understanding of the border line between dermal and endochondral bones obscure; the two types of bones are more similar in terms of their ontogenetic history and characters than we expected. However, the current data has not been so abundant as to be conclusive. For example, the information of cell origins of dermal and endochondral bones in pectoral fin development are still fragmentary. Complete understanding of the cell lineages with genetic labeling in each ossification in wild-type and genetic mutants would provide us an opportunity to propose a more reliable model for the fin-to-limb transition. The most recent studies have elegantly showed that we are able to track the entire cell lineages of early vertebrate development (Briggs et al., 2018; Farrell et al., 2018; Wagner et al., 2018). Comparative analysis of the cell lineages of appendages between fish and tetrapods by deploying these state-of-the-art techniques would help us better our understanding of cell history in appendage development. Also, explicit understanding of genetic basis of dermal and endochondral interchanges needs a significant amount of future work. Previous studies have revealed molecular mechanisms of dermal and endochondral ossifications, yet, the genetic switches to determine dermal and endochondral is still obscure—one of the most critical questions in this field. The integrative approach of high-throughput comparative sequencing, such as RNA-sequencing or ChIP-sequencing, and subsequent functional tests would be a powerful means to discover the genetic loci that have been responsible for dermal-to-endochondral transitions.

Assimilating knowledge from the different structures would lead us one step closer to understanding the whole picture of the vertebrate skeletal shifts. Though the details of genetic mechanisms that regulate dermal and endochondral bones are disparate in each structure, the principal components in bone differentiation, such as *Bmp, Sox9, Runx2* are clearly conserved. The distinct developmental programs in the skull, the shoulder, and fins are likely to be explained by genetic modifications or deployment of different molecules through a long journey of ancestral vertebrates. Future elaboration of molecular mechanisms in distinct types of ossification in multiple structures will shed light on the common and derived genetic mechanisms of dermal and endochondral development.

Embedding the knowledge about the developmental programs of bones into the background of paleontology with newly emerging tools, shifts our understanding of vertebrate evolution into a new era. Reevaluation of the mechanisms underlying the major skeletal shifts in vertebrates with genomics, genetics, and imaging techniques will cast a new light on the deep history of our ancestors.

## Author contributions

TN served as the main author of the body of the manuscript. He also wrote the figure captions as well as completing parts the figure illustrations. TW drafted and made significant edits to the main content of the text, as well as editing the figure captions. He also contributed to the illustration of the figures.

### Conflict of interest statement

The authors declare that the research was conducted in the absence of any commercial or financial relationships that could be construed as a potential conflict of interest.
